# Azithromycin induces epidermal differentiation and multivesicular bodies in airway epithelia

**DOI:** 10.1186/s12931-019-1101-3

**Published:** 2019-06-24

**Authors:** Ari Jon Arason, Jon Petur Joelsson, Bryndis Valdimarsdottir, Snaevar Sigurdsson, Alexander Gudjonsson, Skarphedinn Halldorsson, Freyr Johannsson, Ottar Rolfsson, Fredrik Lehmann, Saevar Ingthorsson, Paulina Cherek, Gudmundur H. Gudmundsson, Fridrik R. Gardarsson, Clive P. Page, Olafur Baldursson, Thorarinn Gudjonsson, Jennifer A. Kricker

**Affiliations:** 1 Stem Cell Research Unit, BioMedical Center, School of Health Sciences, University of Iceland, Reykjavík, Iceland; 2 Center for Systems Biology, University of Iceland, Reykjavík, Iceland; 3 Department of Laboratory Hematology, Landspitali-University Hospital, Iceland, Reykjavík, Iceland; 4 Department of Anatomy, Faculty of Medicine, University of Iceland, Reykjavík, Iceland; 5 BioMedical Center, Life and Environmental Sciences, University of Iceland, Reykjavík, Iceland; 6 EpiEndo Pharmaceuticals, Reykjavík, Iceland; 7 Department of Respiratory Medicine, Landspitali-University Hospital, Reykjavík, Iceland; 8 Sackler Institute of Pulmonary Pharmacology, Institute of Pharmaceutical Science, King’s College London, London, UK

**Keywords:** Azithromycin, Air-liquid interface, Gene expression, Airway, Epithelia, Epidermal differentiation

## Abstract

**Background:**

Azithromycin (Azm) is a macrolide recognized for its disease-modifying effects and reduction in exacerbation of chronic airway diseases. It is not clear whether the beneficial effects of Azm are due to its anti-microbial activity or other pharmacological actions. We have shown that Azm affects the integrity of the bronchial epithelial barrier measured by increased transepithelial electrical resistance. To better understand these effects of Azm on bronchial epithelia we have investigated global changes in gene expression.

**Methods:**

VA10 bronchial epithelial cells were treated with Azm and cultivated in air-liquid interface conditions for up to 22 days. RNA was isolated at days 4, 10 and 22 and analyzed using high-throughput RNA sequencing. qPCR and immunostaining were used to confirm key findings from bioinformatic analyses. Detailed assessment of cellular changes was done using microscopy, followed by characterization of the lipidomic profiles of the multivesicular bodies present.

**Results:**

Bioinformatic analysis revealed that after 10 days of treatment genes encoding effectors of sterol and cholesterol metabolism were prominent. Interestingly, expression of genes associated with epidermal barrier differentiation, *KRT1, CRNN, SPINK5* and *DSG1*, increased significantly at day 22. Together with immunostaining, these results suggest an epidermal differentiation pattern. We also found that Azm induced the formation of multivesicular and lamellar bodies in two different airway epithelial cell lines. Lipidomic analysis revealed that Azm was entrapped in multivesicular bodies linked to different types of lipids, most notably palmitate and stearate. Furthermore, targeted analysis of lipid species showed accumulation of phosphatidylcholines, as well as ceramide derivatives.

**Conclusions:**

Taken together, we demonstrate how Azm might confer its barrier enhancing effects, via activation of epidermal characteristics and changes to intracellular lipid dynamics. These effects of Azm could explain the unexpected clinical benefit observed during Azm-treatment of patients with various lung diseases affecting barrier function.

**Electronic supplementary material:**

The online version of this article (10.1186/s12931-019-1101-3) contains supplementary material, which is available to authorized users.

## Background

The global burden of chronic lung diseases has been demonstrated in various epidemiological studies. Patients with lung diseases including COPD, asthma, diffuse panbronchiolitis and cystic fibrosis (CF) are often admitted to hospitals with acute exacerbations [1]. These admissions can be due to underlying bacterial infections for which the first line of treatment includes macrolide antibiotics [2–8]. Patients that receive macrolide therapy show improved prognosis with fewer and less severe acute exacerbations resulting in fewer hospital admissions [8–11]. These beneficial effects appear to be independent of bactericidal activity [12] and are particularly shown to improve health in patients with COPD regardless of the status of the airway bacterial infections, including that of *P. aeruginosa* colonization [2, 13, 14].

Macrolides are compounds whose chemical structure includes a macrolactone ring backbone. The first identified macrolide was the 14-membered erythromycin. [15, 16]. Azithromycin (Azm), a 15-membered macrolide derived from erythromycin [17], is one of the most prescribed antibiotics in the U.S. [18] and is known to have additional effects aside from its primary role as an antibiotic. Meta-analysis of patients receiving Azm for treatment of chronic airway diseases reveals that many of these patients have fewer acute exacerbations that require hospital admissions. Traditionally macrolides are said to be anti-inflammatory and capable of modulating inflammatory responses, in addition to their bactericidal effect [19]. However, this hypothesis is debated, and the pharmacological activities that explain the observed clinical benefit of Azm remain unproven. Indeed, a recent clinical study reported that Azm reduced exacerbations when administered for 48 weeks to patients with asthma. Interestingly, sputum samples neither indicated significant anti-microbial activity, nor changes in the number of inflammatory cells [20].

Complementing these findings, it has been shown that Azm enhances epithelial barrier function of bronchial epithelial cells when cultivated under air-liquid interface (ALI) conditions [21]. Azm increased the transepithelial electrical resistance (TEER) of VA10, a bronchial epithelial cell line, in ALI culture, while also affecting the processing of tight junction proteins. Moreover, epithelial integrity was maintained during exposure of airway epithelia to *P. aeruginosa* infection [22]. In another study by Slater et al. [23], it was demonstrated that Azm increased TEER in human primary bronchial epithelial cells in ALI culture upon challenge with lipopolysaccharides derived from *P. aeruginosa*. They showed that Azm increased the thickness of the epithelium, reduced the mucin production and resulted in a decrease in metalloprotease (MMP9) production. Collectively the data illustrate that AZM has a barrier effect. Further understanding of this unusual and off target effect of Azm is important and could produce novel strategies to combat barrier failure.

Based upon several previous studies we hypothesized that the effect of Azm was primarily on airway epithelial cells. Therefore, we investigated the effects of Azm on epithelial gene expression and found that Azm had pronounced temporal effects on bronchial epithelial cells grown in ALI culture. Gene sequencing analysis demonstrated increasing differentiation towards an epidermal phenotype with upregulation of several skin associated markers. This was accompanied by the formation of multivesicular and lamellar bodies that may be one of the factors that contribute to the barrier enhancing effects of Azm. Exploring further the barrier enhancing effects of Azm is of importance as barrier failure in the respiratory system can contribute to a wide range of disease conditions and exacerbation of chronic airway diseases.

## Material and methods

### Cell culture

VA10 [24] and BCi-NS1.1 [25] (Cornell University Licensing Agreement) cells lines were cultured in BEGM medium supplemented with retinoic acid, penicillin (50 IU/mL) and streptomycin (50 μg/mL) (Invitrogen). VA10 cells were used between passages 11–20, and BCi-NS1.1 cells between passages 11–24. Both cell lines were maintained in a humidified incubator at 37 °C and 5% CO_2_. Medium was changed every 2 to 3 days.

### Air-liquid interface culture

Air-liquid interface (ALI) culture was conducted using both VA10 and BCi-NS1.1 cells. Transwell filter inserts (Corning 0.4 μM PET membrane) were pre-coated with human type IV collagen (Sigma), and cells were trypsinized and seeded in 50:50 DMEM/Ham’s F-12 containing 10% FBS (ThermoFisher). 2.0 × 10^5^ cells/cm^2^ and 4.5 × 10^5^ cells/cm^2^ of VA10 and BCi-NS1.1, respectively, were seeded in 0.5 mL on the apical side of the insert, and 1.5 mL medium added to the basolateral chamber. After 1 day, the medium in both chambers was changed to DMEM/Ham’s F-12 containing 2% Ultroser G (Pall Scientific). After 2 days when the cell layer was confluent, medium was removed from the apical chamber to create an air-interface, and treatments commenced. Typically, treatments were for either 2 or 3 weeks, and medium was changed every 2–3 days. Azm used was Zithromax® (Pfizer, NY, USA) (53.4 µM) and from Sigma (Missouri, USA) (31.8 µM). All experiments were done in technical triplicates, as well as biological replicates.

### Paracellular flux assay and TEER

VA10 cells were grown in ALI culture and sodium fluorescein (Sigma) permeation was performed at different time points to determine the paracellular flux from the apical to basolateral compartments. ALI cultures were equilibrated for 30 min in Hanks’ Balanced Salt Solution (Gibco HBSS). Sodium fluorescein in HBSS (50 μM) was added to the apical chamber, with 1.5 mL of HBSS in the basolateral chamber. Sampling from the basolateral compartment was done after 20, 40, 60, 80 and 120 min and HBSS volume replenished each time.

TEER was measured before and after the experiment to ensure barrier integrity.

### RNA sequencing and gene expression analysis

RNA was extracted from cells grown in ALI culture and poly-A mRNA libraries were prepared for deep sequencing analysis using an Illumina HiSeq 2500. Triplicate samples were prepared and sequenced from Azm treated cells and untreated cells at days 4, 10 and 22.

The Kallisto (v 0.43) program [26, 27] was utilized for the measurement of differential expression and to obtain q values for each transcript. For enrichment analysis we selected transcripts with differential expression in Azm treated cells versus untreated cells based on Kallisto/sleuth q-values, and with at least two-fold increases in expression (beta > 0.63). The top differently expressed transcripts based on q-values (q < 0.01) (for day 22: 1000 upregulated transcripts and bottom 500 downregulated transcripts) were analyzed with the Panther classification system for GO biological processes (The PANTHER (protein annotation through evolutionary relationship) classification system (version 183), released on 12 September, 2018; http://www.pantherdb.org/) [28] to identify biological processes up or down regulated in the Azm treated cells (Overrepresentation Test).

### qRT-PCR

Total RNA was extracted with Tri-Reagent (Ambion, ThermoFisher). One μg of RNA was reverse transcribed with hexanucleotide primers using Superscript IV (ThermoFisher). Resulting cDNA was used as template for qPCR. Primer pairs and probes from Applied Biosystems (TaqMan) were used for *CRNN* (Hs.PT.58.45584843), *DSG1* (Hs.PT.58.19323131), *KRT1* (Hs.PT.58.24741966), *SPINK5* (Hs.PT.58.27676526), and *PPIA* (Hs.PT.39a.22214851) or *GAPDH* (Hs.PT.39a.22214836) as endogenous reference genes. Gene expression is performed in triplicate technical replicates, as well as biological replicates.

### Immunostaining

Samples were fixed in formalin and embedded in paraffin before being sectioned and immunostained. Three μm thick slides were incubated at 60 °C for an hour prior to staining. Before staining, the sample antigen retrieval was done in a buffer with either citric buffer (pH 6) or TE buffer (pH 9) for 20 min. Samples were then blocked with serum. The primary antibody was incubated overnight at 4 °C and the secondary antibody incubated at room temperature for 30 min. Secondary antibodies used included Dako EnVision+ system-HRP labelled polymer anti-mouse (K400011–2), anti-rabbit (K400211–2) and the DAB substrate kit (ab94665). A kit containing DAB chromogen and substrate buffer (ab94665) was used according to the manufacturer’s instructions.

### Microscopy

Immunofluorescence was visualized and captured using an Olympus FV1200 Confocal microscope (Olympus, Tokyo, Japan). Bright-field and phase-contrast images of samples were captured using an EVOS FL Auto 2 imaging system (ThermoFisher).

### Transmission electron microscopy

VA10 and BCi-NS1.1 cells were grown as monolayers on coverslips and under ALI conditions, and prepared for electron microscopy. Cells were fixed with 2.5% glutaraldehyde for 20 min. Fixed coverslips/filters were placed in 2% osmium tetroxide, followed by a phosphate buffer rinse. Cells were dehydrated and then embedded in resin. 100 nm sections were cut with an Ultramicrotome (Leica EM UC7). Sections were stained with lead citrate (3%, J.T. Baker Chemical Co.) and imaged using a JEM-1400PLUS PL Transmission Electron Microscope.

### LipidTOX assay

Cells were grown with and without treatment of Azm (53.4 μM) for 5 days. The cells were then seeded into 96 well plates containing 1X LipidTOX™ phospholipidosis detection reagent (ThermoFisher) and incubated for 72 h and live cell imaged using an FV1200 Olympus Inverted Confocal Microscope. Quantification of fluorescence intensity was measured using ImageJ. A total of three biological repeats were done (*n* = 3).

### Lipidomic analysis

Lipids were extracted from cell cultures by liquid-liquid extraction. Thawed cell pellets were resuspended in ice-cold methanol, vortexed vigorously and allowed to stand on ice for 10 min. Equal amounts of water and chloroform were added to a final composition of 1:1:1 (CH_3_OH:H_2_O:CHCl_3_), vortexed and left to stand overnight at 4 °C. The organic phase was collected into a glass vial and solvent evaporated in a miVac concentrator (SP scientific, Warminster PA, USA) and reconstituted 2-propranol:ACN:H_2_O (2:1:1, v/v/v) for analysis.

Lipidomic analysis was performed using ultra performance liquid chromatography (UPLC, ACQUITY, Waters, Manchester, UK) coupled with a quadrupole time-of-flight mass spectrometry (Synapt G2, Waters, Manchester, UK) operating in MS^E^ mode with ion mobility separation. Samples were injected onto a 1.7 μm particle 100 × 2.1 mm CHS C18 column (ACQUITY, Waters, Manchester, UK). Mobile phase A was water/acetonitrile 80:20 (v/v) containing 0.05% formic acid and 5 mM ammonium formate, mobile phase B was 2-propanol/acetonitrile 90:10 (v/v) containing 0.05% formic acid and 5 mM ammonium formate. Data acquisition took place over the mass range of 150–1100 Da with two alternating scan modes: a low energy mode with the collision energy in the trap cell set at 6 eV, and a high energy mode with the collision energy in the trap cell set at 6 eV and a collision energy ramp ranging from 20 to 30 eV in the transfer cell. In both scan modes the scan time was 0.5 s. Data processing and analysis were performed with MassLynx v4.1 (Waters), Targetlynx v4.1 (Waters) and Driftscope v2.8 (Waters). Statistical analysis was performed with Metaboanalyst as previously described [29].

### Statistical analysis

All growth curves were performed in triplicate for statistical accuracy. Graphs were created in GraphPad Prism. Statistical significance was determined using Student’s t-tests. Error bars represent the standard deviation (SD) of the sample.

## Results

### Azm treatment of human bronchial epithelial cells increases transepithelial resistance and reduces paracellular flux

**Fig. 1:**
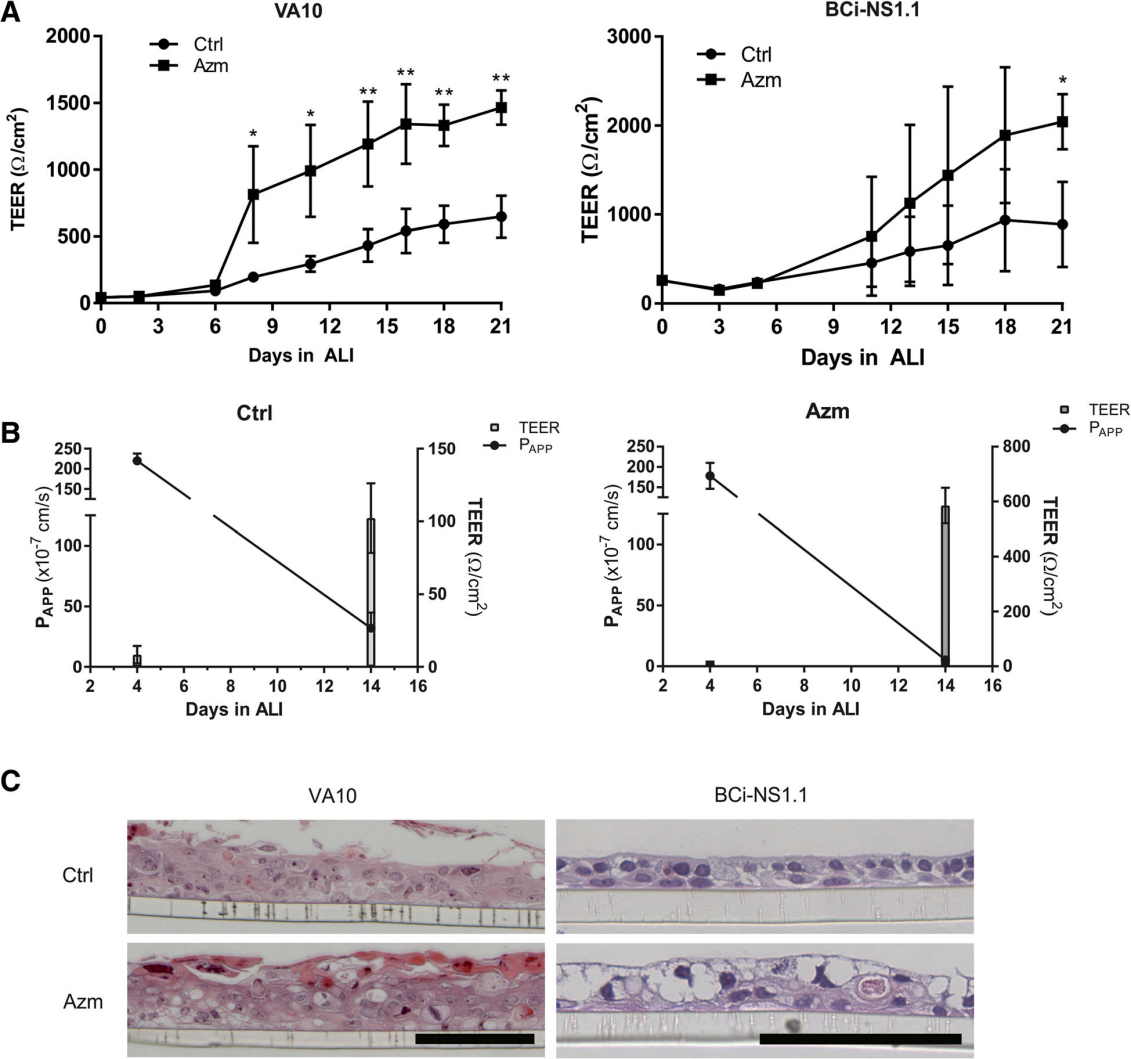
Azm treatment enhances TEER and decreases paracellular flux of airway epithelium culture in ALI. **a**) Azm increases TEER in bronchial-derived basal epithelial cell lines. VA10 (top left) and BCi-NS1.1 (top right). Cells were treated with 25 μg/ml (Azm) for 3 weeks, and then RNA was harvested. Bars represent the average from triplicate wells. Significant differences between Ctrl and Azm are indicated (*P* < 0.05*; *P* < 0.01**). **b**) The apical to basolateral permeability of airway epithelia decreased over time in both control and Azm treated cells. Apparent permeability of sodium fluorescein (Na-Flu was measured as an indication of paracellular flux in VA10 cells. This was inversely correlated with an increase in TEER. **c**) Azm treatment produces a thicker cell layer in airway epithelia. Representative cross-sectional images of VA10 (left) and BCi-NS1.1 (right) cells after culture in ALI for 3 weeks with Azm treatment (bottom) and without (top). Note the increase in thickness of the epithelial layer and formation of vacuoles in Azm treated cells. Cells were counterstained with hemotoxylin. Bar = 100 μm

We first sought to confirm the effect of Azm on bronchial epithelial cells from previous studies. Cells were grown in ALI conditions for 3 weeks and TEER and paracellular flux were measured. Here, we show that both the VA10 and the bronchial-derived basal cell line BCi-NS1.1 [25] demonstrate an inverse relationship between TEER and paracellular flux after treatment with Azm. After approximately 1 week of Azm treatment, TEER begins to increase as compared to non-treated controls (Fig. 1a). Conversely, as TEER increases, paracellular flux decreases (Fig. 1b). In monolayer culture, the VA10 cell line shows a P63/Cytokeratin 14 positive basal epithelial phenotype [30] and thus, the ALI cultures can be considered “naïve” at day 0. To examine if Azm treatment resulted in histology alterations in the ALI cultures, ALI filters were embedded in paraffin and cross-sectioned for microscopic analysis. We observed a thickening of the epithelial layer and the formation of large intracellular vesicles with Azm treatment (Fig. 1c).

### Azm treatment of bronchial epithelial cells results in global and temporal changes in gene expression

**Fig. 2:**
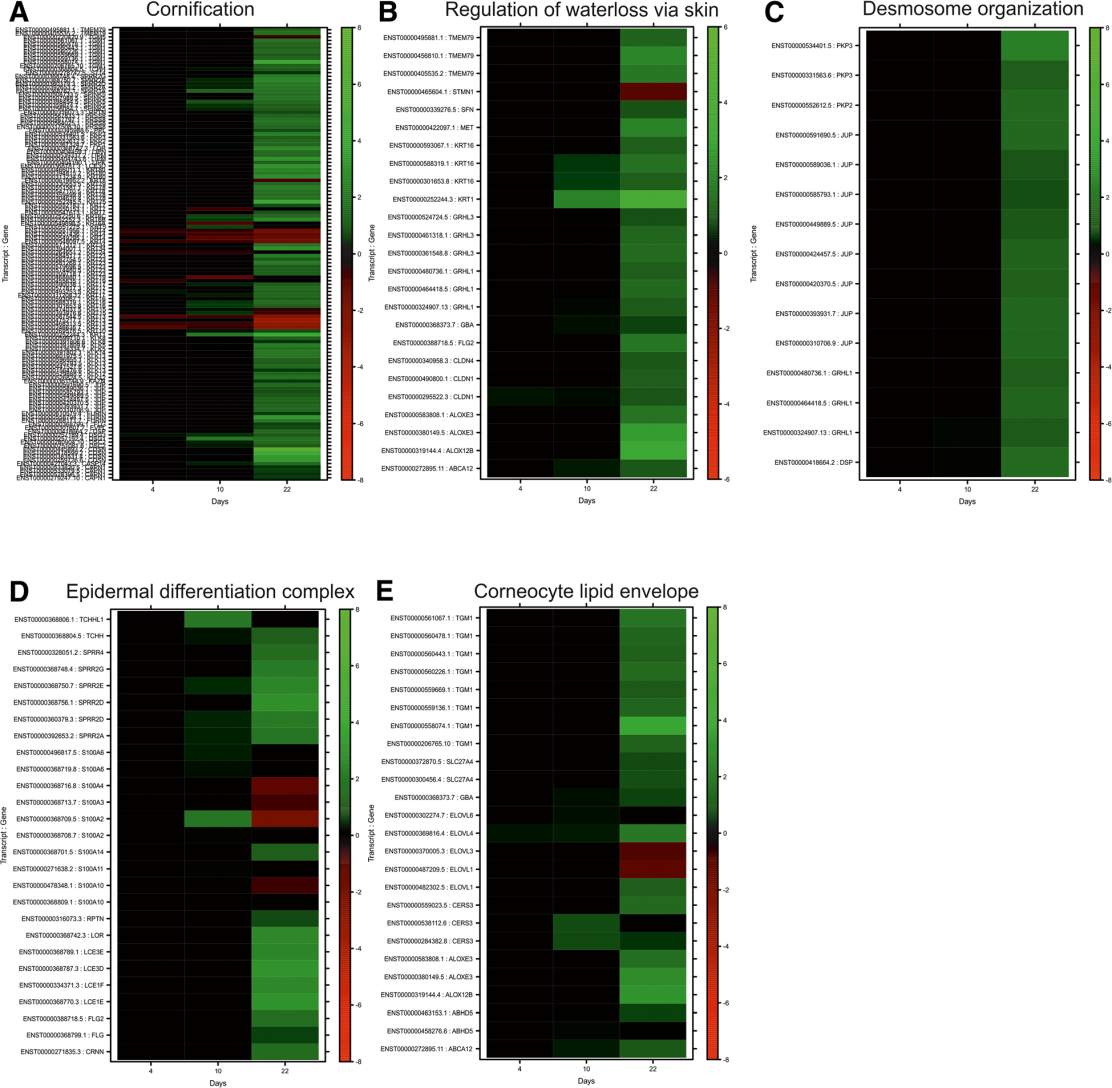
Heatmaps showing upregulated genes involved in late epidermal differentiation and barrier formation as a result of Azm treatment of airway epithelia. Analysis of RNA sequencing of VA10 cells after 4, 10 and 22 days of treatment of Azm in ALI culture. Sequencing was done on triplicate samples. Heatmap showing hierarchically clustered log2(ratio) data, where the ratio is defined as mRNA expression level in control cells to Azm-treated cells. Data are shown for genes differentially expressed at one or more of the three time points during differentiation. Heatmaps showing up/downregulation of transcripts arranged in gene ontology (GO)-groups involved, including (**a**) cornification, (**b**) regulation of water loss via skin, (**c**) desmosomal organization, (**d**) epidermal differentiation complex (EDC), (**e**) corneocyte lipid envelope (CLE). Green color represents increased gene expression relative to red color representing decreased expression

In order to investigate the scope of gene expression changes associated with the cellular observations after Azm treatment in VA10 cells during ALI culture, RNA sequencing was performed on samples collected at three time points during culture: day 4, 10 and 22. The time points selected reflect various stages of differentiation as indicated by rising TEER and reduced paracellular flux. This extended duration of treatment from 2 weeks to 3 weeks was based on reports stating that long term Azm treatment is beneficial for patients with chronic airway diseases [4, 31]. Gene expression analysis revealed global changes throughout the culture period. To assist in interpreting these data, we used the PANTHER gene ontology (GO) classification system [32] to identify striking patterns in the development of increased epithelial barrier function. At day 4, only a few gene transcripts (36) were upregulated according to our statistical criteria (q-value less than 0.01 and more than 2-fold upregulation). However, at day 10, the expression profile was enriched in genes associated with sterol/cholesterol metabolism, an important component of epidermal barrier formation (Table 1) [33–35]. Interestingly, after 22 days of treatment, an overexpression profile in the domain of epidermal ontology groups was enriched. This included keratinization, cornification, keratinocyte differentiation, and establishment of skin barrier regulation of water loss via skin and desmosome organization. The 20 most upregulated genes and their assigned gene ontology groups at the last time point were analyzed and a strong epidermal barrier fingerprint was evident (Table 2). Of particular interest we saw a significant upregulation of *NDRG1*, which recently was directly associated with positive effects on airway epithelial barrier strengthening [36]. We then set out to depict the differential expression pattern of gene transcripts within these categories during Azm treatment and included transcripts known to be active in the formation of the skin barrier related to corneocyte lipid envelope and the epidermal differentiation complex (EDC). The EDC is a complex consisting of proteins encoded by 51 genes involved in the terminal differentiation and cornification of keratinocytes, spanning a 1.9 Mb stretch within chromosome 1q21. After 4 days of treatment, there was very little effect on expression within these categories. After 10 days there was low to intermediate upregulation, while after 22 days a clear upregulation pattern was distinct (Fig. 2a-e). Since transcripts can be classified into more than one GO group, we utilized a recent publication to increase our confidence that these upregulating effects were truly epidermal related. Gerber et al. [37] revealed 687 genes as being specifically skin associated genes (SAG) and assigned them to a total of 15 functional groups. Our results revealed that of the statistically upregulated genes induced by Azm treatment on day 22, 40.3% pertained to the SAG group, in contrast to 14.8% of an equally numbered list of genes that were randomly generated. Interestingly, one of the three most represented functions was vesicle formation.

**Table 1:** Azm induces global gene expression changes in airway epithelia

Day 4			Day 10			Day 22		
GO biological process complete	fold enrichment	raw *P*-value	GO biological process complete	fold enrichment	raw *P*-value	GO biological process complete	fold enrichment	raw *P*-value
negative regulation of platelet activation (GO:0010544)	47,39	5,25E-05	positive regulation of receptor catabolic process (GO:2000646)	56,36	6,21E-05	desmosome organization (GO:0002934)	17,34	8,23E-06
ovulation (GO:0030728)	44,9	6,06E-05	intracellular cholesterol transport (GO:0032367)	25,05	4,10E-05	establishment of skin barrier (GO:0061436)	15,9	2,49E-09
negative regulation of hemostasis (GO:1900047)	23,21	3,57E-05	intracellular sterol transport (GO:0032366)	23,48	5,09E-05	regulation of water loss via skin (GO:0033561)	14,45	5,36E-09
negative regulation of blood coagulation (GO:0030195)	23,21	3,57E-05	cellular response to sterol (GO:0036315)	22,1	6,23E-05	cornification (GO:0070268)	9,55	4,19E-22
negative regulation of coagulation (GO:0050819)	21,87	4,45E-05	cellular response to low-density lipoprotein particle stimulus (GO:0071404)	20,88	7,55E-05	intermediate filament cytoskeleton organization (GO:0045104)	6,15	1,71E-05
positive regulation of blood vessel diameter (GO:0097755)	20,68	5,47E-05	nitric oxide mediated signal transduction (GO:0007263)	19,78	9,07E-05	intermediate filament-based process (GO:0045103)	6,02	2,01E-05
alpha-amino acid catabolic process (GO:1901606)	14,22	3,39E-05	low-density lipoprotein particle clearance (GO:0034383)	19,57	1,20E-05	multicellular organismal water homeostasis (GO:0050891)	5,96	1,29E-06
cellular amino acid catabolic process (GO:0009063)	11,95	7,50E-05	cholesterol biosynthetic process (GO:0006695)	17,89	4,91E-08	water homeostasis (GO:0030104)	5,95	5,17E-07
positive regulation of vasculature development (GO:1904018)	11	4,31E-06	secondary alcohol biosynthetic process (GO:1902653)	17,48	5,77E-08	keratinization (GO:0031424)	5,14	1,29E-15
negative regulation of cell activation (GO:0050866)	9,43	5,00E-05	regulation of cholesterol biosynthetic process (GO:0045540)	17,48	5,77E-08	peptide cross-linking (GO:0018149)	4,9	9,40E-05
regulation of vasculature development (GO:1901342)	7,29	1,61E-05	regulation of sterol biosynthetic process (GO:0106118)	17,48	5,77E-08	skin development (GO:0043588)	4,7	1,78E-21
regulation of angiogenesis (GO:0045765)	7,03	7,03E-05	isoprenoid biosynthetic process (GO:0008299)	16,2	2,69E-05	regulation of epidermis development (GO:0045682)	4,64	1,46E-05
regulation of small molecule metabolic process (GO:0062012)	6,44	3,81E-05	sterol biosynthetic process (GO:0016126)	15,66	1,23E-07	keratinocyte differentiation (GO:0030216)	4,64	2,76E-15
small molecule biosynthetic process (GO:0044283)	5,78	1,13E-06	plasma lipoprotein particle clearance (GO:0034381)	14,09	8,41E-06	epidermal cell differentiation (GO:0009913)	4,37	1,05E-15
negative regulation of cell proliferation (GO:0008285)	5,05	4,49E-06	regulation of cholesterol metabolic process (GO:0090181)	13,42	3,56E-07	regulation of sodium ion transport (GO:0002028)	4,32	2,87E-05
regulation of cell migration (GO:0030334)	4,54	5,33E-06	interferon-gamma-mediated signaling pathway (GO:0060333)	13,23	1,34E-08	cell-cell junction organization (GO:0045216)	4,07	9,97E-07
regulation of cell motility (GO:2000145)	4,25	1,08E-05	regulation of alcohol biosynthetic process (GO:1902930)	12,87	1,71E-08	epidermis development (GO:0008544)	4,02	2,52E-17
regulation of locomotion (GO:0040012)	3,91	2,57E-05	regulation of steroid biosynthetic process (GO:0050810)	12,45	4,44E-09	cellular response to decreased oxygen levels (GO:0036294)	3,83	1,22E-06
regulation of cellular component movement (GO:0051270)	3,89	2,72E-05	regulation of plasma lipoprotein particle levels (GO:0097006)	11,74	1,84E-07	negative regulation of immune effector process (GO:0002698)	3,74	3,32E-05
secretion by cell (GO:0032940)	3,78	3,62E-05	regulation of steroid metabolic process (GO:0019218)	11,74	6,70E-11	cellular response to hypoxia (GO:0071456)	3,66	7,17E-06
regulation of immune response (GO:0050776)	3,52	7,42E-05	cholesterol transport (GO:0030301)	11,74	4,39E-06	cellular response to oxygen levels (GO:0071453)	3,46	4,92E-06
regulation of immune system process (GO:0002682)	3,12	2,23E-05	cholesterol metabolic process (GO:0008203)	10,9	7,51E-10	cell junction organization (GO:0034330)	3,44	3,69E-07
immune system process (GO:0002376)	2,76	7,14E-07	secondary alcohol metabolic process (GO:1902652)	10,44	1,22E-09	epithelial cell differentiation (GO:0030855)	3,16	1,17E-16
regulation of developmental process (GO:0050793)	2,53	2,87E-05	sterol transport (GO:0015918)	9,96	1,18E-05	regulation of autophagy (GO:0010506)	3,11	7,36E-08
regulation of response to stimulus (GO:0048583)	2,02	5,62E-05	cholesterol homeostasis (GO:0042632)	9,76	3,21E-06	protein autophosphorylation (GO:0046777)	3,05	4,19E-05

GO annotation raw *p* value <0.0001

List of 25 GO categories statistically overrepresented for overexpressed genes at each time point analyzed (day 4, 10 and 22) after comparing Azm treatment to non-treated ALI cultures

**Table 2:** An epidermal barrier fingerprint is seen after 22 days of Azm treatment

Transcript ID	q-value	Fold enrichment	Gene ID	Gene name	GO categories
ENST00000252244.3	3,69E-115	3,90	KRT1	Keratin, type II cytoskeletal 1	peptide cross-linking, keratinization, neutrophil degranulation, establishment of skin barrier, cornification
ENST00000259726.6	5,84E-70	3,21	CDSN	Corneodesmosin	cell adhesion, epidermis development, keratinocyte differentiation, cornification
ENST00000368787.3	5,29E-69	3,07	LCE3D	Late cornified envelope protein 3D	peptide cross-linking, keratinocyte differentiation, cornification
ENST00000514033.1	2,47E-65	2,83	KLF3	Krueppel-like factor 3	negative regulation of transcription from RNA polymerase II promoter, multicellular organism development
ENST00000301659.8	7,11E-62	2,78	GSDMA	Gasdermin-A	apoptotic process
ENST00000425231.2	3,37E-61	3,34	CDCP1	CUB domain-containing protein 1	protein binding, extracellular region
ENST00000246635.7	1,08E-60	-2,77	KRT13	Keratin, type I cytoskeletal 13	cytoskeleton organization, keratinization, cornification
ENST00000395254.7	1,08E-60	6,04	ZNF365	Protein ZNF365	telomere maintenance,protein binding,cytoplasm
ENST00000369091.6	1,10E-57	7,37	PRDM1	PR domain zinc finger protein 1	negative regulation of transcription from RNA polymerase II promoter, positive regulation of gene expression
ENST00000557785.5	1,95E-53	6,85	MEF2A	Myocyte-specific enhancer factor 2A	negative regulation of transcription from RNA polymerase II promoter, apoptotic process, dendrite morphogenesis
ENST00000409458.3	6,14E-52	3,61	GPNMB	Transmembrane glycoprotein NMB	cell adhesion, signal transduction, negative regulation of cell proliferation
ENST00000311946.7	6,12E-51	2,40	NIPAL4	Magnesium transporter NIPA4	magnesium ion transmembrane transporter activity
ENST00000313234.9	5,04E-49	2,81	KRT80	Keratin, type II cytoskeletal 80	keratinization, cornification
ENST00000252252.3	1,03E-48	2,49	KRT6B	Keratin, type II cytoskeletal 6B	cytoskeleton organization, keratinization, cornification
ENST00000335585.9	1,87E-48	2,57	PPP2R2C	Serine/threonine-protein phosphatase 2A 55 kDa regulatory subunit B gamma isoform	protein phosphatase type 2A complex
ENST00000605952.5	1,51E-47	2,32	ATG9B	Autophagy-related protein 9B	autophagosome assembly, protein localization to phagophore assembly site
ENST00000293502.1	8,18E-47	3,11	SDR9C7	Short-chain dehydrogenase/reductase family 9C member 7	retinol dehydrogenase activity, nucleolus
ENST00000244360.6	9,75E-45	2,26	RNF39	RING finger protein 39	cellular_component, cytoplasm
ENST00000395641.2	5,65E-44	2,24	NUPR1	Nuclear protein 1	positive regulation of protein modification process, negative regulation of cell cycle
ENST00000269703.7	1,58E-43	2,20	CYP4F22	Cytochrome P450 4F22	monooxygenase activity,iron ion binding,endoplasmic reticulum membrane
ENST00000377950.7	7,18E-43	2,27	LYPD5	Ly6/PLAUR domain-containing protein 5	extracellular region,plasma membrane
ENST00000224784.10	4,78E-42	-2,29	ACTA2	Actin, aortic smooth muscle	positive regulation of gene expression
ENST00000522476.5	8,87E-42	2,45	NDRG1	Protein NDRG1	negative regulation of cell proliferation, DNA damage response, signal transduction by p53 class mediator, regulation of apoptotic process, cellular response to hypoxia
ENST00000329078.7	1,01E-41	2,17	SPNS2	Protein spinster homolog 2	sphingolipid metabolic process, locomotion
ENST00000393200.6	2,04E-40	2,70	IL36RN	Interleukin-36 receptor antagonist protein	negative regulation of cytokine-mediated signaling pathway,cytokine activity,interleukin-1 receptor binding
ENST00000317216.2	1,66E-39	2,08	EGR3	Early growth response protein 3	positive regulation of endothelial cell proliferation,cell migration involved in sprouting angiogenesis
ENST00000368087.7	5,45E-38	2,83	ARG1	Arginase-1	neutrophil degranulation
ENST00000372789.5	4,313E-37	2,02	WFDC5	WAP four-disulfide core domain protein 5	serine protease inhibitor
ENST00000234396.8	6,879E-35	-2,15	ATP6V1B1	V-type proton ATPase subunit B, kidney isoform	ossification,ATP binding,cytoplasm,cytosol,microvillus
ENST00000447113.6	1,713E-33	2,06	DMKN	Dermokine	keratinocytes, differentiation of epidermis

**Fig. 3:**
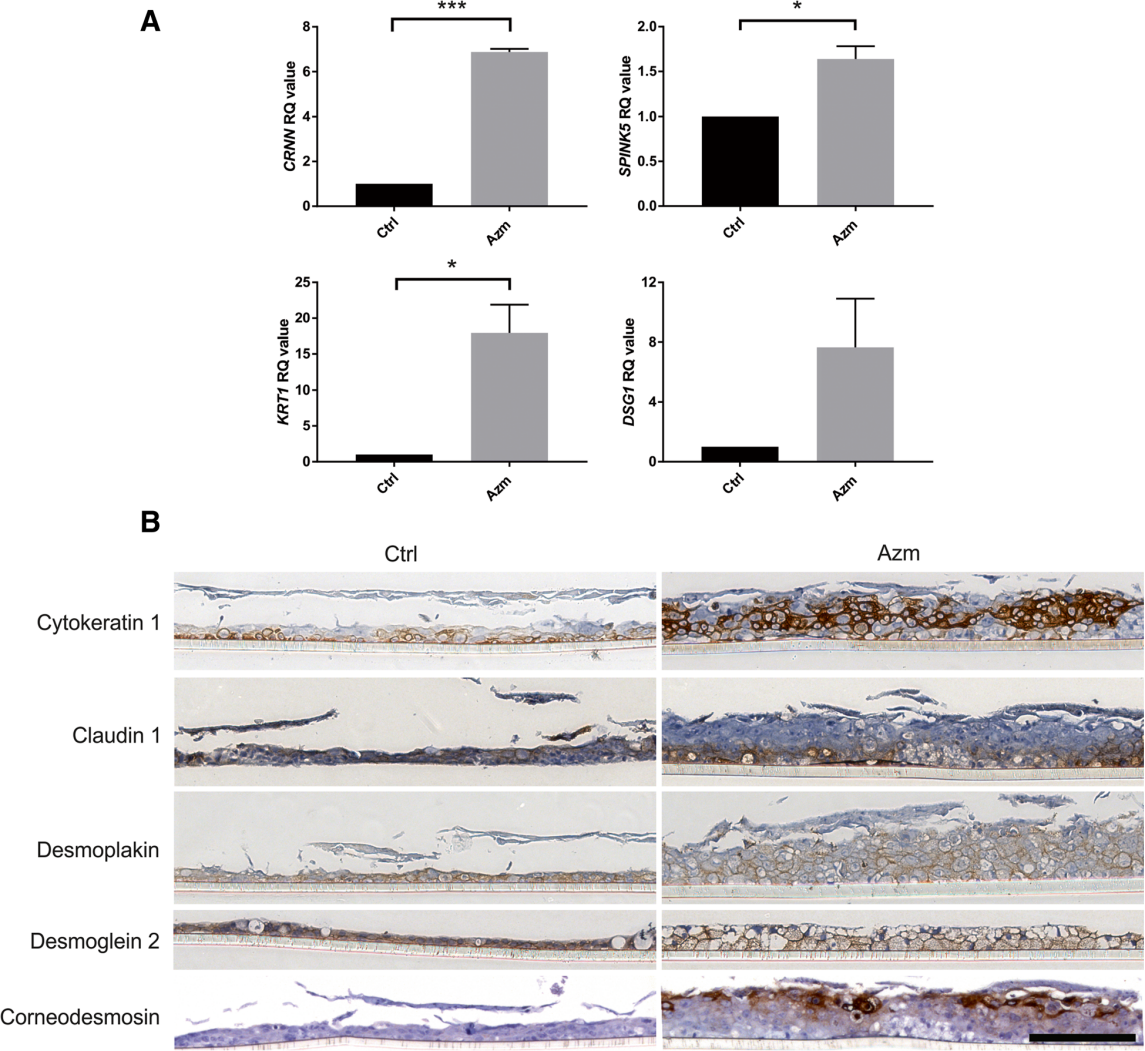
Confirmation of genes and proteins upregulated after Azm treatment. **a**) Expression of four genes identified in the top 30 list (Table 2). Expression was confirmed using qRT-PCR of RNA isolated from 22-day cultures treated with Azm compared to control. RNA was pooled from duplicate wells and each sample was measured in triplicate in qPCR. Data shown is averaged from 3 separate experiments (*P < 0.05; ***P < 0.001). Abbreviations: *CRNN*-cornulin; *KRT1*-cytokeratin 1; *SPINK5*-serine protease inhibitor Kazal-type 5; *DSG1*-desmoglein 1. **b**) Expression of epidermal associated markers in VA10 cells after Azm treatment. ALI cultures from 22 day treatment with Azm were stained using immunohistochemistry for proteins shown to have enhanced gene expression in the sequencing data. Azm-treated samples stained positive for epidermal markers cytokeratin 1, desmoplakin, desmoglein 2 and corneodesmosin, in addition to the tight junction protein claudin 1. In addition to positive staining, notable thickened cell layers were observed after Azm treatment. Cells were counter-stained with hematoxylin. Bar = 100 μm

Expression of a selection of genes from each of the GO groups was confirmed using qRT-PCR (Fig. 3a). Genes showing significant increase after Azm treatment included: Cytokeratin 1 (*KRT1),* that acts together with keratin 10 to form intermediate filaments to provide strength, albeit most notably in skin; Cornulin (*CRNN*), also known as squamous epithelium shock protein 53 that is suggested to play a role in the mucosal/epithelial immune response and epidermal differentiation; and desmoglein 1 (*DSG1*), a major component of desmosome cell-cell junctions. Additionally, we also observed changes in the serine protease inhibitor Kazal-inhibitor 5 (*SPINK5*), which is known to regulate proteases and be involved in respiratory processes. To assess any alterations in the tissue architecture, cross-sections of ALI cultures from day 22 were examined (Fig. 3b). Notably, the Azm treated cultures were thicker and vesicle formations were apparent. ALI cultures treated for 3 weeks with Azm were stained for markers with increased gene expression. Again, staining of epidermal barrier associated proteins revealed dramatic increase in expression of cytokeratin 1 and corneodesmosin, with notable de novo apical expression of the latter. The tight junction protein claudin 1 along with desmoplakin and desmoglein 2 were also expressed (Fig. 3b).

### Azm treatment induces accumulation of intracellular vesicles containing phospholipids

**Fig. 4:**
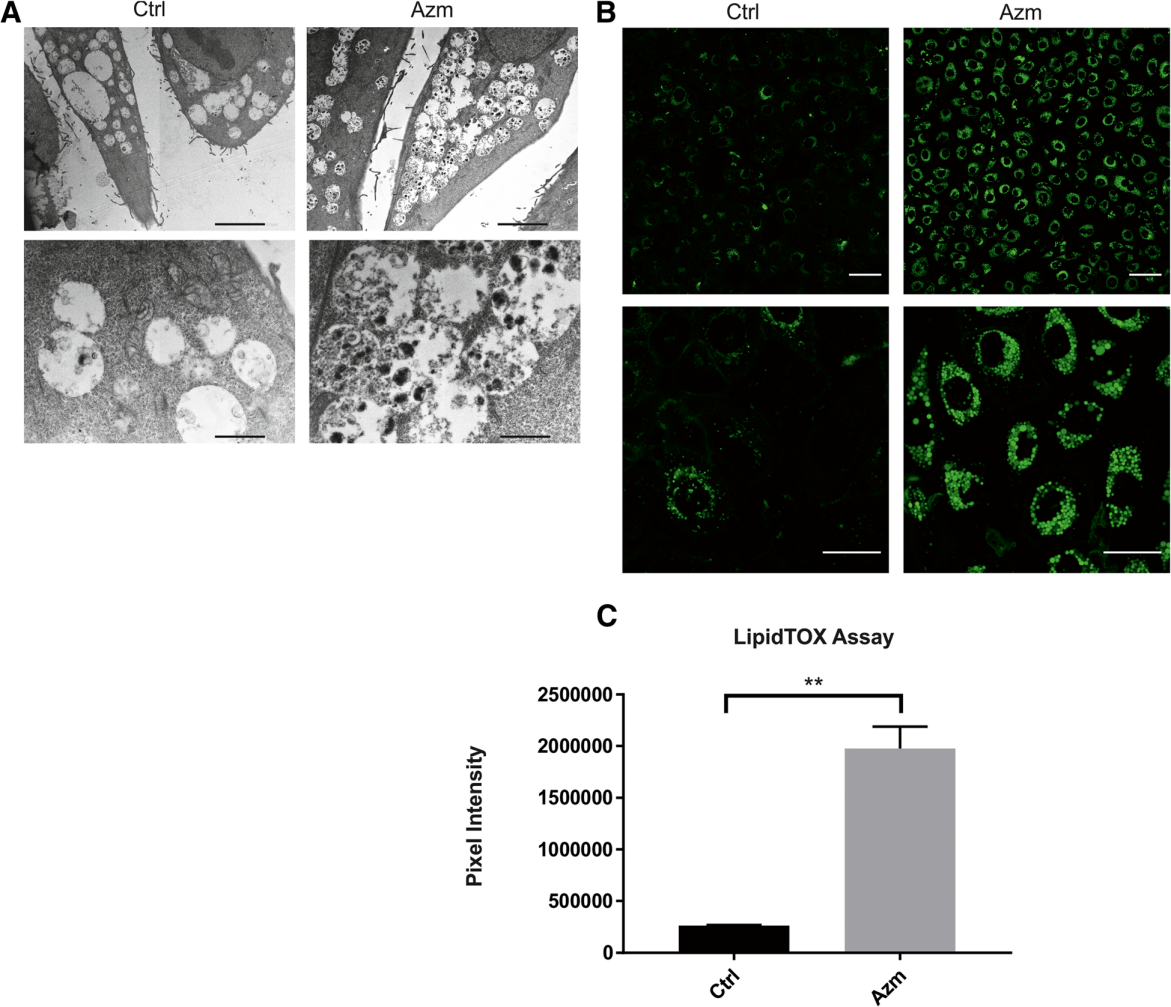
Increased vesicle formation and lipid retention in Azm treated airway epithelial monolayer. **a**. Transmission electron microscope images showing that airway epithelia grown in monolayer (VA10 cells) treated with Azm have substantially more vesicle formation than the controls. Many of these vesicles were identified phenotypically as multivesicular bodies (MVB). Top scale bars are 5.0 μm and bottom scale bars are 1.0 μm. **b**. Increased lipid retention in Azm-treated VA10 cells seeded with HCS LipidTOX reagent. Representative images from 3 biological replicates. Top scale bar is 40 μm and bottom scale bars are 20 μm. **c**. Quantification of LipidTOX retention from confocal microscopy images in Fig. 4b. The mean pixel intensity of different wells from 3 different experiments was calculated using ImageJ (*P* < 0.01)

To further characterize the vesicle formation following Azm treatment, we performed transmission electron microscopy imaging on airway epithelia grown in monolayer. Both VA10 and BCi-NS1.1 cells treated with Azm for 5 days showed a clear increase in vesicle formation and many of these vesicles were phenotypically identified as multivesicular bodies (MVB) (Fig. 4a and Additional file 1: Figure S1). These vesicles were shown to accompany lipid accumulation as highlighted by a lipidTOX assay, whereby phospholipids conjugated to a fluorescent dye are added to the culture media (Fig. 4b). The Azm treated cells showed a significant (*P* < 0.01) increase in lipid retention when compared to the control (Fig. 4c).

### Formation of multivesicular- and lamellar- bodies with Azm treatment in ALI cultures

**Fig. 5:**
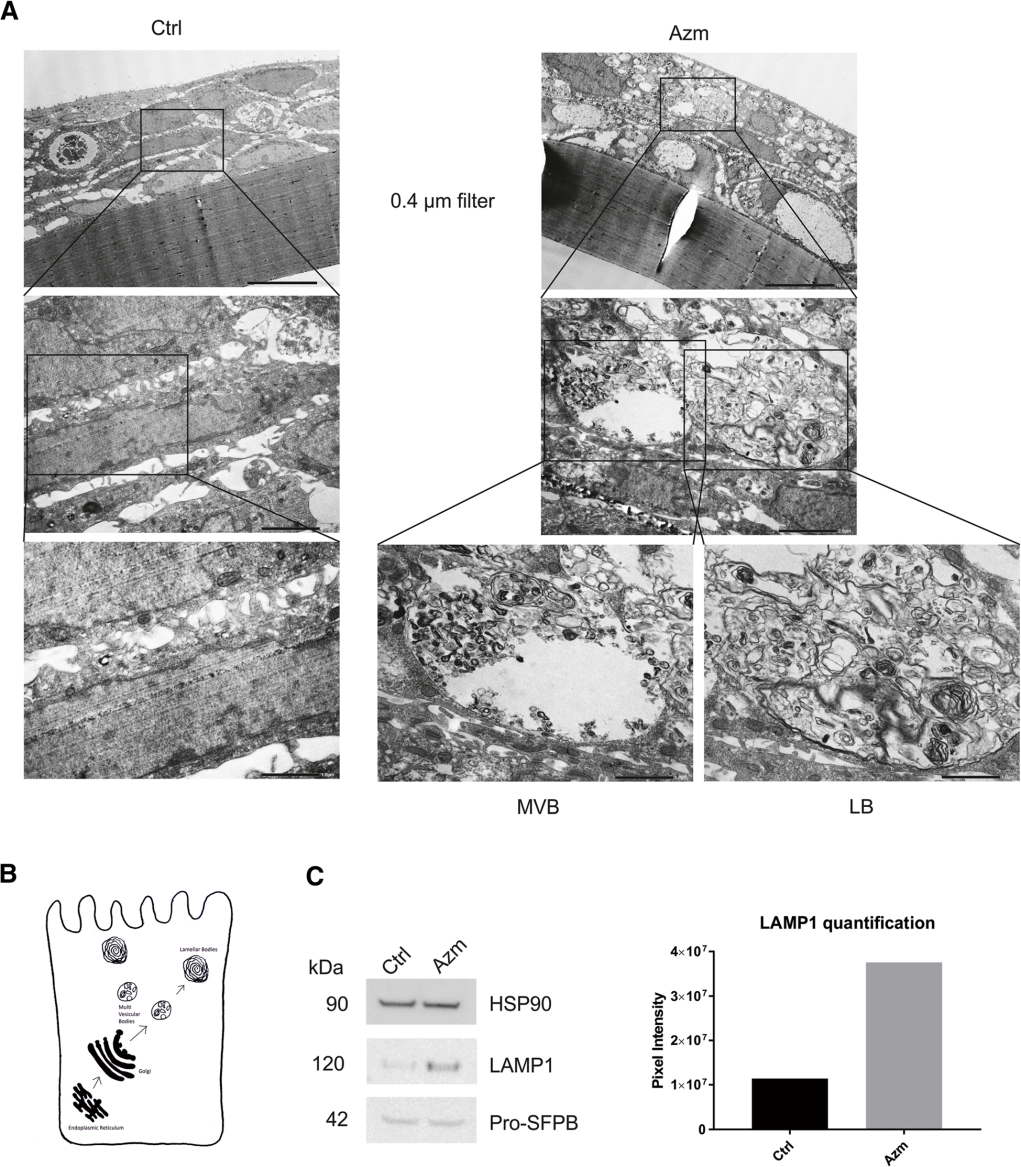
Formation of epidermal barrier – Azm induced lamellar body formation. **a**. VA10 cells differentiated on 0.4 μm filters in ALI cultures treated with Azm (Panel far right) showed a marked increase in MVB formation. LB formations were identified in the cells but not present in the control cells (panel far left). Images show different magnifications. Scale bars are 10.0 μm for top images, 2.0 μm for middle images and 1.0 μm for bottom images. **b**. Simplified illustration of LB formation based on Brasch et al. 2004 [38]. **c**. LAMP1 protein expression is increased in Azm treated cells while Pro-SFPB expression is unchanged. LAMP1 quantification is seen on the right

Combining the data from the epidermal GO groups and the lipid-containing vesicles after Azm treatment raised the question as to whether the MVB identified in Fig. 4a mature into lamellar bodies (LB). Cells cultured in ALI conditions and treated with Azm showed the same MVB formations as the Azm treated monolayer cells as seen in cross sectional electron microscope images (Fig. 5a). LB formations were observed in these cell layers. This was not seen in any of the ALI cultured control cells (Fig. 5a and Additional file 2: Figure S2A). LB formations were also prevalent in ALI cultured cells treated with a clinical formulation of Azm (Zithromax) (Additional file 2: Figure S2B). This was not limited to VA10 cells, as the BCi-NS1.1 cell line displayed similar MVB and LB formation after Azm (Zithromax) treatment (Additional file 2: Figure S2C). Figure 5b (adapted from [38]) summarizes the formation of MVB and LB. Western blot analysis for known markers related to LBs revealed an increased expression in LAMP1 (lysosome-associated membrane glycoprotein 1) [39] in Azm treated cells, while pro-surfactant protein B (Pro-SFPB) did not (Fig. 5c).

### Lipid-Azm conjugates accumulate inside cells

**Fig. 6:**
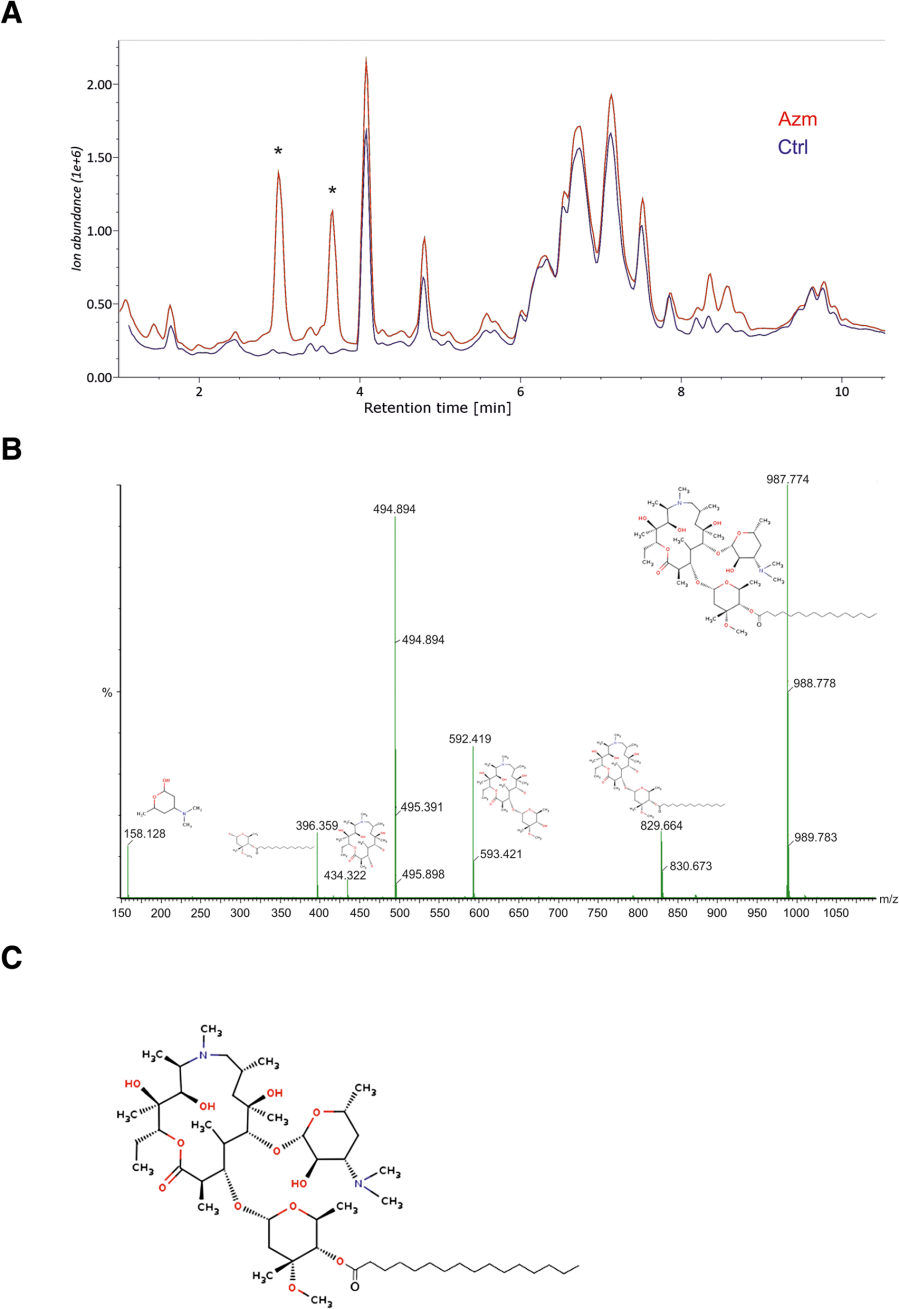
Conjugation of fatty acids to Azm in treated airway epithelia. **a**) Total ion chromatograms of representative samples of control (blue) and Azm treated VA10 cells (red). Large peaks appear at 2.99 and 3.66 min in the Azm treated cells. **b**) Fragmentation of these peaks (2.99 peak shown here) shows many similarities to the fragmentation pattern of Azm but with an m/z shift of 239.3 in some of the fragments. This corresponds precisely to condensation of palmitate to one of the side-groups of AZM while releasing H_2_O. A similar pattern, with a shift of m/z = 267.3 was seen in the fragmentation of the 3.66 peak. This corresponds to condensation of stearate to Azm. **c**) Chemical structure of palmitate - Azm conjugate

To further analyze the lipid retention of the Azm treated cells, non-polar metabolites were extracted from VA10 and BCi-NS1.1 cells grown in monoculture and VA10 cells cultured in ALI conditions, with and without Azm treatment. Lipid extracts were analyzed with UPLC-MS. Ion chromatograms of Azm treated VA10 cells cultured in monolayer showed two large unidentified peaks at 2.99- and 3.66-min retention times that were not present in the untreated cells (Fig. 6a). Ion spectra of these peaks show that they contained fragment patterns similar to Azm but without the native form at m/z = 750. Unknown compounds of m/z = 239 (2.99 peak) and m/z = 267 (3.66 peak) appear to be conjugated to Azm to create compounds of m/z = 988 or m/z = 1016, respectively (Fig. 6b). These charge-mass ratios correspond to condensation of saturated fatty acids, specifically palmitate (C16:0) and stearate (C18:0) to Azm (Fig. 6c). A smaller peak representing Azm conjugation to myristic acid (C14:0) was also detected. Azm-lipid conjugates were detected in high quantities in both VA10 cells and BCi-NS1.1 cells treated with Azm. These Azm-lipid conjugates were also present in VA10 ALI cultures treated with Azm for 3 weeks, although these changes were not as pronounced.

**Fig. 7:**
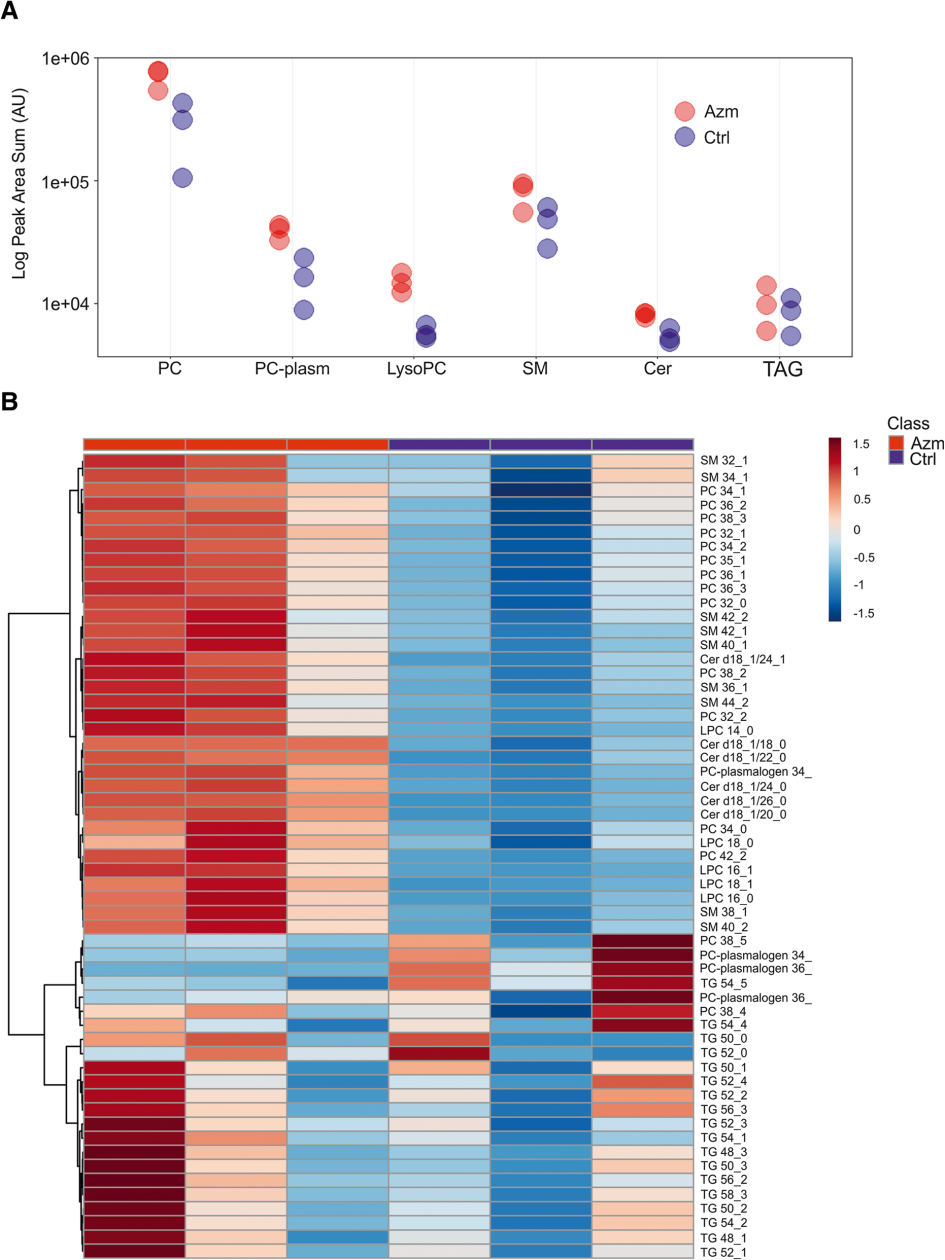
Lipid composition of Azm treated airway epithelia during prolonged ALI culture. **a**) Relative abundance of 6 lipid classes in Azm treated (red) and untreated cells (blue). **b**) Heatmap of the 50 compounds that demonstrate the largest differences between Azm treated and untreated cells. Values were normalized by total lipid intensity

Targeted analysis of 57 lipid species of six lipid classes showed accumulation of phosphatidylcholine (PC) species and lyso-phosphatidylcholine (lysoPC) in Azm treated ALI cultures, as well as ceramide derivatives (ceramide and sphingomyelin (SM)) (Fig. 7a). Figure 7b depicts a heatmap of all lipids identified in the analysis. In general, most of the PCs are higher in all the Azm treated samples than any of the control samples. The only exceptions are the long-chain poly-unsaturated PCs; PC 38:4 and PC 38:5 that are either not different between the two groups or higher in untreated cells. All lysoPCs analyzed here are more pronounced in the Azm treated cells than the untreated cells. In contrast, with the exception of PC-plasmalogen 34:0, the plasmalogens detected were found to be reduced in Azm treated cells compared to untreated cells. All SM species detected were more abundant in Azm treated cells as were most of the ceramide species. The only exception was ceramide 18:1/16:0 which was higher in the untreated cells. Of the 18 triacylglycerol (TAG) species, all but one were found at similar levels between Azm treated and control cells. The exception was TAG 54:5 that was found to be higher in the untreated cells. This was the TAG species with the highest level of unsaturation having five double-bonds.

Taken together, Azm appears to be conjugated to saturated fatty acids upon entering the cell. The direct functional consequences of this are unknown. During differentiation of the cells during ALI, Azm treated cells maintain far higher levels of structural phospholipids such as PC and SM while storage lipids such as TAG are unaffected.

## Discussion

We have previously shown that Azm increases TEER in a bronchial-derived basal epithelial cell line, VA10, when cultured in ALI conditions [21]. Here, we show that Azm induces an increase in TEER that corresponds to a decrease in paracellular flux, in both the VA10 and the bronchial-derived basal cell line BCi-NS1.1 [25].This suggests that Azm treatment of cultured airway epithelial cells results in increased integrity of an epithelial barrier in culture and enhanced barrier functions. Furthermore, we have demonstrated that Azm treatment alters the global gene expression pattern over time in airway epithelial ALI cultures. Ten days of Azm treatment results in increased lipid metabolism, which complements microarray study findings from Ribeiro et al. [40], who found that Azm treatment of human bronchial epithelial (HBE) cells resulted in upregulated lipid / cholesterol metabolism after 24 h of treatment. In the skin, lipid-rich lamellar bodies (LB) are first seen in the spinous layer, accumulate in the granular layer and finally form the intercellular lamella of the stratum corneum. The intercellular lamellae contain cholesterols, along with phospholipids and glucosylceramides, and together these lipids create a hydrophobic layer that is key in the permeability barrier of the skin [41]. Here, we have observed initial alterations associated with lipid remodeling and metabolism at day 10, with an increased expression pattern towards epidermal differentiation extending to day 22. Within the epidermal gene expression data set, subclasses such as desmosome-related genes, corneocyte lipid envelope and cornification, are highly enriched. Conceivably related to this epidermal phenotype shift, we found the formation of MVB and ultimately, LB. LB formation in the lungs has traditionally been associated with type 2 alveolar cells, but has recently been reported in bronchial epithelial cells [42]. Club cells also express surfactant proteins (A, B, C and D) [43]. In the lungs, LB derived from MVB are lipid-protein transporters, important in the transporting of surfactant proteins to the lumen of the alveoli and vital to epidermal barrier formation [38]. Pro-surfactant B (Pro-SFPB) is processed into its mature form from the Golgi complex via the MVB into LB, which in turn transport the surfactant to the cell membrane for excretion [38] (Fig. 5c). In the epidermis, LB are formed in the trans-Golgi network and their main function is to secrete and deliver lipids (mostly cholesterol, glucosylceramides, phospholipids and sphingomyelin) to the extracellular reaches of the stratum corneum, crucial for the integrity of the permeability/water barrier. The finding that Azm induces the expression of various subclasses within epidermal differentiation in bronchial epithelial cells and enhances the epithelial barrier is of interest, not only for the respiratory epithelium but possibly for other epithelial layers as well. Increased TEER in ALI cultured lung cells and thus enhanced barrier effect has been explained by greater tight junction expression [21]. Our results indicate that a contributing factor to increased TEER could be lipid accumulation in cells, as fatty tissues are among the most resistant of human tissues [44].

The results presented here indicate that the induction of epidermal differentiation genes following treatment with Azm causes formation of MVB and LB. This is accompanied by thickening of the cell layer and sequestering of Azm-lipid conjugates into intracellular droplets that may partly be responsible for barrier enhancement. Azm is a cationic amphiphilic drug and as such is a known inducer of phospholipidosis [45]. In a recent article, Liu et al. argued that drug induced phospholipidosis, which has historically been viewed as an adverse side-effect of macrolide treatment, could be beneficial under certain circumstances [46]. These authors demonstrated that Azm-induced phospholipidosis brought about accumulation of LB in human Meibomian gland epithelial cells promoting their function. We propose that Azm is having a similar effect in the airway epithelium. Azm has been shown to improve disease burden of patients with idiopathic pulmonary fibrosis [47] and CF, making it essential to better understand the mechanisms involved in order to discover and produce more specific and relevant pharmaceuticals aimed at patients with these debilitating diseases.

Azm has been of clinical interest due to its immunomodulatory properties and the reports of it leading to a reduction in chronic airway disease exacerbations. Attempts have been made to analyze alterations in gene expression of airway epithelium subsequent to low dose Azm treatment, mostly focusing on downregulated genes [48]. The targeted research for the immunomodulatory mechanism has dominated the field and other possible mechanisms have been largely neglected. We herein, report on the temporal involvement of lipid-cholesterol metabolism and cornification resulting in a strengthened epithelial barrier.

Azm displays beneficial effects in chronic airway disease patients, but as it is an antibiotic, extended clinical use of Azm has the potential to facilitate pathogen drug resistance. A more effective drug in the treatment of these patients would be a non-antibiotic derivative of Azm with anti-inflammatory and epithelial barrier enhancing effects. It is anticipated that our results will assist in the development of non-antibiotic derivatives of Azm to enhance epithelial barrier function as a novel approach to the treatment of respiratory diseases.

## Conclusions

The data provided herein contribute to our understanding of the mode of action of Azm in the treatment of respiratory diseases and confirm that Azm is more than an anti-microbial drug. Via mechanisms involving lipid metabolism and upregulation of distinct ontologies of genes pertaining to epidermal differentiation, Azm strengthens epithelial airway barriers. The barrier enhancing effects of Azm in vitro are likely a consequence of a combination of events that occur during long term treatment and which results in multiple intracellular changes, which require deeper investigation.

## Additional files

Additional file 1: **Figure S1**. Azm treatment of BCi-NS1.1 cells leads to increased vesicle formation Transmission electron microscope images show that BCi-NS1.1 cells treated with Azm have substantially more vesicle formation than the untreated controls. Left scale bars are 10.0 μm and right scale bars are 1.0 μm. (TIF 18391 kb)

Additional file 2: **Figure S2**. Azm induced MVB and LB formation. A) BCi-NS1.1 cells differentiated in ALI cultures treated with Azm showed a marked increase in MVB and LB formations. Shown are two different cross sectional TEM images of transwell filters. Scale bars are from left 10.0, 2.0, 1.0 and 1.0 μm. B) Treating differentiated VA10 cells with a clinical formulation of Azm (Zithromax) also resulted in increased MVB and LB formations. Top scale bars are 5.0 μm and bottom scale bars are 1.0 μm. C) Differentiated BCi-NS1.1 cells showed similar MVB and LB formations after treatment with Azm (Zithromax). Top scale bars are 5.0 μm and bottom scale bars are 1.0 μm. (TIF 33306 kb)

## Data Availability

RNA sequencing data with accession number GSE128523 will be publicly available at GEO. Other data are included within this manuscript.

## Abbreviations

ACN: acetonitrile
ALI: air-liquid interface
Azm: azithromycin
CF: cystic fibrosis
EDC: epidermal differentiation complex
GO: gene ontology
HBE: human bronchial epithelial
HBSS: Hanks’ balanced salt solution
LB: lamellar bodies
MMP: metalloprotease
MVB: multivesicular bodies
PC: phosphatidylcholine
SAG: skin associated genes
SFPB: surfactant protein B
SM: sphingomyelin
TAG: triacylglycerol
TEER: transepithelial electrical resistance
